# TWO-LEVEL CONFIRMATORY FACTOR ANALYSIS OF THE POSITIVE AND NEGATIVE AFFECT SCHEDULE (PANAS) IN MID- AND LATE LIFE

**DOI:** 10.1093/geroni/igae098.3513

**Published:** 2024-12-31

**Authors:** Madeline Nichols, John Geldhof

**Affiliations:** Oregon State University, Corvallis, Oregon, United States; Oregon State University, Corvallis, Oregon, United States

## Abstract

The Positive and Negative Affect Schedule (PANAS; Watson et al., 1988) is widely used in the study of well-being; however, its factor structure continues to be a source of debate (Wedderhoff et al., 2021). The present study examined the factor structure of the PANAS using data from the third wave of the National Study of Daily Experiences (NSDE; N=1176, Mage=62.48, SD=10.20, Range=43-91, %Female=57.38) in which participants indicated how much of the time they felt positive and negative emotions each day for eight days. We conducted two-level confirmatory factor analyses in MPlus informed by the 45-degree rotation hypothesis (Yik et al., 1999) that classifies emotion words by both valence (negative or positive) and activation (high or low). Results revealed that a five-factor structure at both the within and between levels exhibited good fit (χ2(188) = 989.76; p <.001; RMSEA = 0.02; CFI = 0.960; TLI = 0.949). These factors were: high activation negative (upset, angry, frustrated); negative (worthless, sad, hopeless, lonely); high activation positive (enthusiastic, attentive, proud, active, confident); positive (happy, cheerful); and low activation positive (calm, satisfied). Latent correlations indicated factor separation at both the within- and between-person levels (median absolute correlations of.44 and.60, respectively). These results emphasize the importance of considering both valence and activation in the study of daily affective well-being. The authors recommend that researchers using the PANAS consider the implications of valence and activation when examining associations between affect and health-related outcomes.

